# Scientific Items

**Published:** 1888-05

**Authors:** 


### SCIENTIFIC ITEMS.

**Who is Never Crazy?**—There are many firm believers in the theory that most people are crazy at times, and facts seem to support their belief. The following, from a source unknown to the writer, will likely remind a number of our readers of some incident in their experience, which at the time of its occurrence seemed to them most unaccountable:

"A wise man will step backward off a porch or into a mud puddle, a great philosopher will hunt for the specks that are in his hand or on his forehead, a hunter will sometimes shoot himself or his dog. A working girl had been feeding a great clothing knife for ten years. One day she watched the knife come down slowly upon her hand. Too late she woke out of her stupor with one hand gone. For a few seconds her mind had failed, and she sat by her machine a temporary lunatic and had watched the knife approach her own hand. A distinguished professor was teaching near a canal. Walking along one evening in summer he walked as deliberately into the canal as he had been walking along the path a second before. He was brought to his senses by the water and mud and the absurdity of the situation. He had on a new suit of clothes and a new silk hat, but though the damage was thus great, he still laughs over the adventure. Our mail collectors find in the iron boxes along the streets all sorts of papers and articles which have been put in by some hand from whose motions the mind has become detached for a second. A glove, a pair of spectacles, a deed, a mortgage, a theater ticket, goes in, and on goes the person, holding on to the regular letter which should have been deposited. This is called absent-mindedness, but is a brief lunacy."—*Scientific American.*

**Simple Methods for Reviving Unconscious Persons.**—At a meeting of the last congress of German scientists this subject was discussed, and Dr. H. Frank mentioned that there are but two ways to stimulate the heart; electricity and mechanical concussion of the heart. The first is considered dangerous by him, as it may easily destroy the last power of contraction remaining in the organ; but what is termed "pectoral concussion" is decidedly preferable. F.'s method is as follows: He flexes the hands on the wrist to an obtuse angle, places them both near each other in the ileocæcal region, and makes vigorous strokes in the direction of the heart and of the diaphragm. These strokes are repeated from fifteen to twenty times, and are succeeded by a pause, during which he strikes the chest over the heart repeatedly with the palm of his hand. In favorable cases this method is early successful, and some times a twitching of the lids or the angles of the mouth appears with surprising rapidity as the first sign of returning life. As soon as the symptoms are noted, the simple manipulations above described must be earnestly continued, and persevered in for from a half to one hour, for with their cessation the phenomena indicating beginning return of life also ceases. Generally the face assumes a slight reddish tint, and at the same time a faint pulsation may be felt in the carotids. By this method Frank has seen life return in fourteen cases, amongst whom were such as had hung themselves, drowned, and asphyxiated by carbonic oxide, and in one case by croup.—*Phrenological Journal.*

**Light from the Stars.**—It has been found by experiments on the light emitted by the stars of different order of magnitude that the light of a star of the sixth magnitude amounts to only one-hundredth part of the light of a star of the first magnitude. Hence we conclude (always supposing the stars to be of equal magnitude and splendor) that a star of the sixth magnitude is ten times more remote than a star of the first magnitude. Now the bright star Alpha Centauri may be considered as typical of a star of the first magnitude. Combining our knowledge of the relative distances of Alpha Centauri and stars of the sixth magnitude with the conclusions above arrived at, it follows that if Alpha Centauri were transported to 750 times it actual distance, it would still be visible in Herschel's 20 foot reflector, and consequently there might be perceptible in such an instrument a star the distance of which is 750 times greater than the actual distance of Alpha Centauri. Now the absolute distance of Alpha Centauri from the earth, as ascertained by the researches of various astronomers, may be stated in round numbers to be 20,000,000,000 of miles. Hence we arrive at the conclusion that the distance of the stars which are faintly visible in a 20-foot reflecting telescope, such as Herschel employed in his observations, is not less than 15,000,000,000 of miles. Light which traverses space with a velocity equal to 186,-000 miles in a second, would occupy more than 2,000 years in passing from such a star to the earth. Well might Herschel remark that the visibility of a star in the present day is proof—not of its actual existence, but rather of its having existed for hundreds or thousands of years ago.—*Phrenological Journal.*

**The Sinking Cordillera of the Andes.**—The Cordillera of the Andes has for some time been exhibiting a curious phenomenon. It results from observations made upon the altitudes of the most important points, that their height is gradually diminishing. Quito, which in 1745 was 9,596 feet above the level of the sea, was only 9,570 feet in 1803, 9,567 in 1834, and scarcely 9,520 feet in 1867. The altitude of Quito has, therefore, diminished by 76 feet in the space of 122 years. Another peak, the Pichincha, has diminished by 218 feet during the same period, and its crater has descended 425 feet in the last twenty-five years. That of Antisana has sunk 165 feet in 64 years.—*Lagazette Geographique.*

**Remote Stars.**—There are some stars so remote that their light, though traveling with a velocity of 186,000 miles per second, cannot reach us in less than 10,000 years. To traverse the Milky Way, of which our own solar system forms a part, light requires 15,000 years; and to reach us from some of the distant nebulæ, which appear like faint clouds, and are hardly visible through the largest telescope, it must travel for 300 times that period, or nearly 5,000,000,000 years.—*Lenoard's Illustrated Medical Journal.*

